# Is Seprafilm valuable in infant cardiac redo procedures?

**DOI:** 10.1186/s13019-015-0257-2

**Published:** 2015-03-31

**Authors:** Bruno Lefort, Jean-Marc El Arid, Anne-Lorraine Bouquiaux, Nathalie Soulé, Julie Chantreuil, Elsa Tavernier, Alain Chantepie, Paul Neville

**Affiliations:** 1 Children Hospital Gatien de Clocheville, Tours, France; 2 University François Rabelais, Tours, France; 3 INSERM, CIC 202, Tours, France

**Keywords:** Congenital heart disease, Cardiac reoperation, Tissue adhesives, Biomaterials

## Abstract

**Background:**

Morbidity and mortality are higher for cardiac reoperations than first operation due to the presence of post-operative adhesions. We retrospectively evaluated the efficacy of the bioresorbable membrane Seprafilm® to prevent pericardial adhesions after cardiac surgery in a paediatric congenital heart disease population.

**Methods:**

Seventy-one children undergoing reoperations with sternotomy redo and cardiopulmonary bypass for congenital malformations were included. Twenty-nine of these patients were reoperated after previous application of Seprafilm® (treatment group). The duration of dissection, aortic cross clamping and total surgery were recorded. A tenacity score was established for each intervention from the surgeon’s description in the operating report.

**Results:**

In multivariate analysis, the duration of dissection and the tenacity score were lower in the treatment than control group (p < 0.01), independent of age and interval since preceding surgery.

**Conclusion:**

Our results suggest that Seprafilm® is effective in reducing the post-operative adhesions associated with infant cardiac surgery. We recommend the use of Seprafilm® in paediatric cardiac surgery when staged surgical interventions are necessary.

## Background

Many children with congenital heart disease require several surgical interventions to achieve correction or palliation. The opening of the pericardial cavity during each operation promotes the formation of postoperative intra-pericardial adhesions. These adhesions complicate reoperation and potentially lead to injury of cardiac structures; consequently, cardiac reoperation is associated with greater morbidity and mortality [1-3]. Moreover, adhesions can lead to dissection being very stressful and time-consuming for surgeons, even before cardiac repair has been started.

Some potentially useful methods to reduce adhesions are now available for clinical use. Obviously, minimally invasive primary operations, perfect haemostasis, drainage of shed blood and clots, and direct closure of the autologous pericardium when possible, are all desirable and minimise adhesion; also the application of an adhesion barrier may help prevent adhesive processes and simplify access during subsequent interventions [4].

In our child cardiac surgery unit, we routinely apply a bioresorbable membrane, Seprafilm®, to the dry mediastinum just before sternal closure if reoperation is likely, to facilitate the future dissection. Here, we report a retrospective evaluation of the efficacy of this technique for preventing pericardial adhesions after cardiac surgery in a paediatric population undergoing surgery for congenital heart disease.

## Methods

### Patients

We retrospectively included all children up to 18 years old, who underwent reoperation including sternotomy redo and cardiopulmonary bypass (CPB) for congenital malformations in our hospital from 1998 to 2009. The same surgeon operated on all the children. Patients were excluded if the reoperation was fewer than 10 days after the previous surgery. During the first operation, the decision whether or not to apply the adhesion barrier was made by the surgeon based on his judgement of the likelihood of reoperation. Thus, 71 children were included in the analysis: 29 underwent reoperation after application of the adhesion barrier in the preceding surgery and composed the treatment group; the other 42 children were reoperated without prior adhesion barrier application and composed the control group. Details of the reoperations are listed in Table 1.

**Table 1:** **Reoperations performed in the two groups**

Reoperation	Treatment group (n=29)	Control group (n=29)
Correction for RVOTO	7	13
Valve surgery (aortic, mitral, pulmonary, tricuspid)	5	12
Pulmonary venous obstruction repair	2	2
Systemic venous obstruction repair	0	1
Pulmonary artery banding	1	0
Aortic arch repair	2	1
VSD closure	1	3
ASD closure	1	0
Correction of Fallot and pulmonary atresia with VSD	5	1
Damus procedure	0	1
Right ventricle aneurysm	1	1
Sub aortic stenosis resection	0	5
Glenn procedure	2	2
Coronary bypass	2	0

RVOTO: right ventricular outflow tract obstruction, VSD: ventricular septal defect, ASD: auricular septal defect.

### Adhesion barrier

A bioresorbable membrane composed of sodium hyaluronate and carboxymethylcellulose (Seprafilm®, Genzyme, Cambridge, MA, USA) was used in all patients in the treatment group. Seprafilm® turns into a hydrophilic gel coat approximately 48 hours after placement and separates pericardial surfaces thereby preventing the formation of fibrin bridges that become adhesions [5].

Seprafilm® was applied at the end of the intervention, just before sternal closure, to the dry mediastinum, after meticulous drainage and removal of shed blood and clots. Two drains were placed in the pericardial cavity at the end of operation. To facilitate the application of the membrane and to cover the largest part of the heart, Seprafilm® sheets were cut into six to eight pieces of sizes according to the corpulence of the patient. Each piece was hydrated in physiological solution and was gently laid on the right lateral, diaphragmatic and anterior faces of the heart, and around the aortic root.

### Measurements

The primary endpoint of the study was the dissection time, the time from the skin incision to the starting of CPB; this time was compared between the treatment group and the control group. The dissection time for each intervention was obtained from the operative records book. This value correlates well with the severity of adherences [6], and was chosen of the primary outcome because of its robustness. The period from skin incision to the removal of CPB (total surgery time) was also recorded, as was the duration of aorta clamping (the time between installation and removal of the aortic clamp or aortic cross clamp time).

The secondary endpoint of this study was the tenacity score of adhesions. The tenacity score was graded for each surgical intervention from the surgeon’s description in the operating report as: none, 0; filmy, 1; moderate, 2 and dense, 3.

Age at reoperation and interval between the preceding surgery and reoperation were also recorded.

### Statistics

Quantitative variables are described as medians and quartiles. Parametric models were used including potentially confounding factors. Non parametric Mann and Whitney tests were used to compare age at reoperation and interval from the preceding surgery to reoperation between the two groups. Dissection time, total operation time and aortic clamping time were analysed by multiple linear regression. The severity of adherences was analysed by multinomial logistic regression.

### Ethical issues

Institutional review board permission was obtained to report this surgical technique.

## Results

Dissection time and tenacity score were significantly lower in the treatment group as assessed by univariate analysis, whereas aortic cross clamp time and total surgery time did not differ significantly between the two groups (Table 2).

**Table 2:** **Univariate analysis**

Variable	Treatment group (n = 29)	Control group (n = 42)	p
Age at re-intervention (months)	21 [7; 33]	68.5 [23.25; 111.2]	<0.01
Interval since preceding surgery (days)	250 [91; 670]	1270 [479.2; 2380]	<0.01
Dissection time (minutes)	47 [38; 81]	86 [65.25; 110.8]	<0.01
Aortic cross clamp time (minutes)	75 [49; 97]	71 [51.5; 99]	0.73
Total surgery time (minutes)	195 [138; 253]	214.5 [183.5; 259.5]	0.30
Adhesion tenacity score			
0	1	2	<0.01
1	21	5	
2	2	12	
3	0	15	

Quantitative variables are reported as medians and quartiles.

Children in the control group were older and intervals from preceding surgery to reoperation were longer than in the treatment group (Table 2). We used multivariate analysis to adjust for these confounding variables. Multiple regression analysis indicated that the application of Seprafilm® in the preceding surgery reduced the dissection time (β = −4e-01, p < 0.01), independently of age and interval from preceding surgery (Table 3). Multinomial logistic regression adjusted for the previously reported confounding factors showed that the use of Seprafilm® was associated with a reduction in the tenacity score (OR = 12.07, p < 0.01) (Table 4). Aortic clamp time and total surgery time did not differ significantly between the two groups.

**Table 3:** **Multiple linear regression analysis of the dissection time**

Dissection time	β Regression coefficient	95% confidence interval	p value
Use of Seprafilm®	−4e-01	[−6e-01; −2e-01]	<0.01
Age at reoperation	2e-03	[−1e-03; 6e-03]	0.21
Interval between surgical interventions	−1e-04	[−3e-04; 5e-05]	0.18

Multiple regression analysis showed that the application of Seprafilm® during the preceding surgery reduced the dissection time independently of age and interval since the preceding surgery.

**Table 4:** **Multinomial logistic regression analysis of tenacity score**

Tenacity score	Odd ratio	95% confidence interval	p value
Use of Seprafilm® during the preceding surgery	12.07	[2.89; 50.49]	<0.01
Age at reoperation	1.00	[0.98; 1.01]	0.61
Interval between surgical interventions	1.00	[0.99; 1.00]	0.26

Multinomial logistic regression analysis showed application of Seprafilm® during the preceding surgery reduced the tenacity score independently of age and interval since the preceding surgery.

## Discussion

We report a significant reduction of the time from skin incision to the starting of CPB (dissection time) in children undergoing repeat sternotomy with the use of Seprafilm® (the primary endpoint of the study). Intuitively, we would expect the duration of this period to be lengthened by the presence of adhesions because they make safe approach to the heart more difficult. Indeed, the length of this period has been shown to correlate with the severity of adherences [6]. We observed the use of Seprafilm® was also associated with a non-significant reduction in the total duration of surgery due to different dissection times, whereas there was no difference in the duration of aortic cross clamp time, which would not be expected to be affected by adhesions. The adhesion tenacity score (the secondary endpoint) was significantly lower in the Seprafilm® group.

There were no injuries to the heart or the major vessels in either group during the study. There is therefore no evidence of whether or not the adhesion barrier improves morbidity or mortality. Nevertheless, the reduction in post-operative adhesions associated with Seprafilm® facilitate and shorten the dissection, and this would be expected to minimise the surgeon’s stress and fatigue before starting the repair of the heart. The beneficial effects of any such phenomenon would be difficult to demonstrate.

Seprafilm® has had European CE mark approval for use in cardiac surgery since 1999, but there have been few analyses of its efficacy to reduce the incidence of adhesions. Walther et al. reported 30 paediatric patients who underwent cardiac reoperation after application of Seprafilm® during the previous surgery: the tenacity score was lower and the duration between incision and the start of CPB was shorter than for ten random control patients [7]. A recent report by Kaneko et al. compared 22 patients who underwent cardiac reoperation after Seprafilm® application during the previous surgery with 23 patients reoperated without application of an adhesion barrier. The surgical adhesions were graded prospectively from video recordings during reoperation: they were less tenacious if Seprafilm® had been used [8].

The clinical use of Seprafilm® in cardiac surgery is recognised as safe [7]. In our study, we did not identify any adverse event such as mediastinum infection or bleeding after application of the membrane. Only one case of delayed healing was observed, and involved a neonate: it was probably due to diffusion of Seprafilm® along the sides of the sternum in this child in who sternal closure was delayed. Particular attention is required during the application of Seprafilm® to avoid this kind of complication. We recommend cutting six to eight appropriately sized pieces of Seprafilm® to cover most of the heart and the origins of the major vessels; this also help prevent the spread of the membrane to the sides of the sternum. Our experience was that use of the membrane was feasible throughout the study, for all ages from neonate to adolescent.

Only four bioresorbable products, one of which is Seprafilm®, have been developed and evaluated in human cardiac surgery to reduce postoperative adhesion. Repel-CV® (SyntheMed Inc, Iselin, NJ, USA) is a polymeric polylactic acid-polyethylene glycol film that, applied to the mediastinum, mechanically separates surfaces [9]. A large prospective multicentre randomised evaluator-masked study showed a lower severity of adhesions in 54 children undergoing a re-sternotomy after application of Repel CV®, than 49 control children with no application of adhesion barrier [6]. Coseal® (Baxter, Deerfield, Il, USA) is a surgical sealant composed of two synthetic polyethylene glycol polymer solutions. Napoleone et al. evaluated the effectiveness of Coseal® sprayed onto the mediastinum after cardiac surgery to reduce the severity of adhesions at seven mediastinum sites [10]. Twenty of the 36 children (56%) who had been reoperated for cardiac congenital disease after previous application of Coseal® had “filmy or avascular” adhesions only, and 31 patients (86%) had “filmy or avascular” adhesions at ≥ 4 sites. The study did not include a control population for comparison. Cova CARD® (Biom’up, Saint Priest, France) is a purified porcine type 1-collagen membrane cross-linked with an oxidized polysaccharide. Armoiry et al. showed non-significantly shorter durations of both dissection and the total process for patients undergoing cardiac reoperation after previous application of Cova CARD® than for a historic reference cohort who did not receive anti-adhesion treatment [11].

### Study limitations

Our study has several biases, some inherent to the retrospective nature of the data collection. The dissection time, directly collected from the operative record book, is probably robust; however, the adhesion score was graded retrospectively from the non-blinded surgeon’s description of adherence in the operating report, and is subjective and possibly inaccurate. Patients were not randomized, and some of the control patients were historical patients included between 1998 and 2001 before Seprafilm® was used in our hospital. Indeed, the children included in this study are heterogeneous. We restricted the analysis to the first reoperation to reduce the diversity of the groups, and we used multivariate analyses to account for confounding factors related to this heterogeneity.

## Conclusion

Despite the possible bias due to the retrospective nature of the study, Seprafilm® appears to be effective at reducing post-operative adhesions in children after cardiac surgery. The product is easy to apply at the end of the surgery, and was found to be safe. In view of these results, we recommend the use of Seprafilm® for paediatric cardiac surgery when staged surgical interventions are necessary.
